# The impact of arthritis and joint pain on individual healthcare expenditures: findings from the Medical Expenditure Panel Survey (MEPS), 2011

**DOI:** 10.1186/s13075-017-1230-3

**Published:** 2017-02-28

**Authors:** Edith M. Williams, Rebekah J. Walker, Trevor Faith, Leonard E. Egede

**Affiliations:** 1 Department of Public Health Sciences, Medical University of South Carolina, 135 Cannon Street, Suite CS303, Charleston, SC 29425 USA; 2 Center for Health Disparities Research, Medical University of South Carolina, 135 Rutledge Avenue, Room 280H, Charleston, SC 29425 USA; 3 Division of General Internal Medicine and Geriatrics, Department of Medicine, Medical University of South Carolina, 96 Jonathan Lucas Street, Suite 803, Charleston, SC 29425 USA; 4 Health Equity and Rural Outreach Innovation Center (HEROIC), Ralph H. Johnson Veterans Affairs Medical Center, 109 Bee Street, Charleston, SC 29401 USA

**Keywords:** Joint pain, Arthritis, Healthcare cost, Medical expenditures, MEPS, Functional limitations

## Abstract

**Background:**

Joint pain, including back pain, and arthritis are common conditions in the United States, affecting more than 100 million individuals and costing upwards of $200 billion each year. Although activity limitations associated with these disorders impose a substantial economic burden, this relationship has not been explored in a large U.S. cohort.

**Methods:**

In this study, we used the Medical Expenditures Panel Survey to investigate whether functional limitations explain the difference in medical expenditures between patients with arthritis and joint pain and those without. We used sequential explanatory linear models to investigate this relationship and accounted for various covariates.

**Results:**

Unadjusted mean expenditures were $10,587 for those with joint pain or arthritis, compared with $3813 for those without. In a fully adjusted model accounting also for functional limitations, those with joint pain or arthritis paid $1638 more than those without, a statistically significant difference.

**Conclusions:**

The growing economic and public health burden of arthritis and joint pain, as well as the corresponding complications of functional, activity, and sensory limitations, calls for an interdisciplinary approach and heightened awareness among providers to identify strategies that meet the needs of high-risk patients in order to prevent and delay disease progression.

## Background

Joint pain (including back pain) and arthritis (osteoarthritis, rheumatoid arthritis) are some of the most common and costly conditions in the United States, affecting more than 100 million individuals and costing more than $200 billion per year [1]. Compared with the general population, those with diseases characterized by arthritis/rheumatism and chronic joint pain experience high rates of functional limitation [1–7]. Functional limitations include those related to limitations in mobility, self-care, daily activities, and pain [1, 3]. Researchers have observed significant impacts to the individual patient as a result of functional limitations, as well as a quantifiable burden to society. One example is the impact of functional limitation on mental health. Turner and Turner [8] examined the relationships among physical disability, mental health, and unemployment. They found that the three factors were closely related and suggested that this combination could lead to a unique pattern of disadvantage for these individuals. They observed that those with disabilities were five times more likely to be involuntarily unemployed and that rates of depression were higher for all individuals with physical limitations, but the trend was more pronounced for those without a job [8]. These unique and compounding factors may significantly increase both healthcare use and associated costs.

Annual direct costs have been estimated on the basis of reported ambulatory care visits, hospitalizations, diagnostic tests, medications, assistive devices, nonallopathic treatments, travel to visits, and paid household help [9]. In other studies focused on specific disorders (i.e., gout, ankylosing spondylitis), researchers have explored costs of illness to the patient and found that functional limitations contribute to such costs and in some cases are the most important predictor of total costs in patients [10, 11].

Indirect costs have been estimated from missed work days or, for retirees and homemakers, the number of days of activity limitation, as well as employment, household income, and receipt of supplemental security income for disability [9, 10, 12]. One investigation revealed that employers lost 28.2 million workdays annually ($4.95 billion in lost income) due to functional limitation caused by chronic diseases, including arthritis [3]. Additionally, 5.3% of the entire U.S. working population experiences work limitations attributable to arthritis [1]. The monetary cost of these work-specific limitations is not yet known, but the prevalence at which these limitations occur may have a significant impact on workplace productivity, especially in physically demanding fields.

Although activity limitations associated with musculoskeletal disorders impose a substantial economic burden on American society, this relationship has not been explored in a large U.S. cohort. As a result, in this study, we used the Medical Expenditures Panel Survey (MEPS) to investigate whether functional limitations explain the difference in medical expenditures between patients with any type or degree of arthritis and/or joint pain and those without.

## Methods

### Sample population

Using the MEPS consolidated file for 2011, we analyzed data from 35,313 adults (ages 18 years and older). Of this sample, 3793 adults self-reported arthritis or joint pain of some type. MEPS is an ongoing national household survey for the civilian noninstitutionalized U.S. population, with oversampling for blacks and Hispanics [13]. The data are collected through in-person interviews and include detailed information on demographic characteristics, self-reported health conditions, health status, use of medical services, charges and sources of payment, access to care, satisfaction with care, health insurance coverage, income, and employment for each person in the household [13]. The complex survey design provides weights to account for sampling and stratification, and it allows generalization to the U.S. population [14, 15]. Use of the complex survey design enables the 35,313 adults in the sample population to represent 311,125,758 individuals living in the United States in 2011. In addition, it allows generalization from the 3793 adults with self-reported arthritis and/or joint pain in the MEPS sample to represent the 150,378,648 population of adults in the United States with arthritis or joint pain.

### Dependent variable

The dependent variable was total healthcare expenditures, defined as the sum of direct payments for office-based medical, hospital inpatient (including zero night stays) and outpatient, emergency department, pharmacy, dental, home health, and other medical care. Total healthcare expenditures represent annual expenditures for the year of reporting (2011) for each individual. Expenditures represent direct payments, not charges, for care provided during 2011 and are collected through both self-report via the household component and through provider reporting via the medical provider component of the survey. The medical provider component is used to verify information collected at the household level, as well as to collect information not known by the household [13].

### Independent variable

The primary independent variable was any self-reported diagnosis with arthritis or joint pain. *Joint pain* and *arthritis* are used here as overarching terms; however, they include any diagnosis of this nature, including various rheumatic conditions as well as back pain. We were also interested in the independent variable “any limitation,” which summarizes whether a person has any limitations in instrumental activities of daily living, activities of daily living, function (walking), activity, or sensory (any visual or hearing impairment). The presence of any limitation was defined as a positive response to any of these components.

### Covariates

Additional covariates were included on the basis of the Anderson model for healthcare use, and we categorized them as predisposing, enabling, and need factors [16]. Predisposing factors included age, race/ethnicity, gender, region, and metropolitan statistical area (MSA). Age was categorized into four groups of 18–34 years, 35–44 years, 45–64 years, and 65+ years. Race/ethnicity was grouped into four categories of non-Hispanic white (NHW), non-Hispanic black, non-Hispanic other (including both Asian and other categories), and Hispanic. Gender was dichotomized. Region was categorized as Northeast, Midwest, South, and West. MSA was dichotomized as MSA (urban) vs. non-MSA (rural).

Enabling factors included household income, employment status, education, insurance, and marital status. Household income was categorized into four groups: <$25,000, $25,000–$49,999, $50,000–$74,999, and > $75,000. Employment was dichotomized. Education was categorized into four groups as less than high school, high school, college, and graduate school. Insurance was categorized into three categories of private, public, and uninsured. Marital status was categorized into three groups of never married, separated/divorced/widowed, and married.

Need factors included health status, body mass index (BMI), and comorbidities. Comorbidities included chronic bronchitis, asthma, cancer, coronary heart disease, myocardial infarction, other heart disease, angina, diabetes, emphysema, high blood pressure, high cholesterol, depression, and stroke. Health status was self-reported into five categories of excellent, very good, good, fair, and poor. BMI was categorized into four groups of underweight, normal, overweight, and obese. Binary indicators of comorbidities were based on a positive response to a question, “Have you ever been diagnosed with ___?,” for each diagnosis.

### Statistical analysis

We used sequential explanatory linear models to investigate whether functional limitations explain the difference in medical expenditures between patients with arthritis and joint pain and those without. For each model, we ran a generalized linear model (GLM) using gamma distribution with total expenditures as the dependent variable and reporting joint pain or arthritis as the main independent variable. We then added variables in blocks according to the Anderson model for healthcare use categories of predisposing variables, enabling variables, and need variables. Finally, we ran a final fully adjusted model and added any functional limitation. After each GLM, we determined the marginal effects using the margins command in Stata 14.0 statistical software (StataCorp, College Station, TX, USA). In order to generalize our study findings to the U.S. population, the complex sampling design of the MEPS dataset was taken into account by using sampling weight, variance estimation stratum, and primary sampling unit in all regression models and sample demographic estimates.

## Results

Table 1 shows the characteristics for U.S. adults with and without arthritis or joint pain. Of the 3793 adults who self-reported any arthritis and/or joint pain, 63% reported any limitations, compared with 18% of individuals not reporting arthritis or joint pain. Unadjusted mean expenditures were $10,587 for those reporting joint pain or arthritis, compared with $3813 for those without. Most (87.7%) of U.S. adults reporting arthritis or joint pain are older than 45 years of age, and they are predominantly NHW (75.6%) and female (63.1%). The majority are not employed (58.1%) and have private insurance (63.1%). The most common comorbidities are high blood pressure (63.3%) and high cholesterol (55.8%), and the majority are overweight (32.2%) or obese (42.6%).

**Table 1:** Sample characteristics of U.S. adults with self-reported arthritis or joint pain (sample = 3791; population = 311,125,758)

	U.S. adults reporting arthritis or joint pain	U.S. adults in sample with no reported arthritis or joint pain
Limitations, %		
Any limitations	62.86	18.18
IADL	9.16	1.80
ADL	5.68	1.03
Walk limitations	39.01	6.12
Mean total expenditure ± SD	$10,587 ± $504	$3813 ± $119
Predisposing factors, %		
Age, years		
18–24	0.77	15.36
25–44	11.54	39.19
45–64	45.85	32.72
65+	41.85	12.72
Race		
Non-Hispanic white	75.64	64.76
Non-Hispanic black	11.66	11.42
Non-Hispanic other	4.77	7.58
Hispanic	7.93	16.24
Sex		
Male	36.86	50.49
Female	63.14	49.51
Region		
Northeast	18.29	18.28
Midwest	23.24	21.07
South	38.48	36.75
West	19.99	23.91
MSA		
Rural	19.17	14.72
Urban	80.83	85.28
Enabling factors, %		
Income		
< $25,000	48.59	42.88
$25,000–$49,999	29.33	29.80
$50,000-$74,999	12.38	14.70
> $75,000	9.70	12.62
Employment		
Not employed	58.05	28.73
Employed	41.95	71.27
Education		
Less than high school	17.34	14.66
High school	31.44	27.70
College	40.87	45.59
Graduate school	10.35	12.05
Insurance		
Any private	63.09	68.99
Public only	30.46	14.67
No insurance	6.45	16.35
Marital status		
Never married	10.48	31.04
Separated/divorced/widowed	35.30	16.61
Married	54.22	52.34
Need factors, %		
Asthma	15.21	7.80
Diabetes	20.11	7.19
Emphysema	7.12	1.21
High blood pressure	63.28	26.05
High cholesterol	55.75	25.01
Stroke	9.89	2.14
Depression	17.32	6.98
CVD	32.32	9.91
BMI		
Underweight	1.45	1.96
Normal	23.80	37.05
Overweight	32.17	34.29
Obese	42.58	26.70

*Abbreviations: ADL* Activities of daily living, *BMI* Body mass index, *CVD* Cardiovascular disease, *IADL* Instrumental activities of daily living, *MSA* Metropolitan statistical area

As shown in Table 2, adults with self-reported arthritis or joint pain pay, on average, $6773 more in medical expenditures than those not reporting arthritis or joint pain. Adjustment decreased the marginal difference to $4427 when accounting for predisposing factors; $3980 when accounting for predisposing and enabling; and $2764 when accounting for predisposing, enabling, and need factors. Finally, in a fully adjusted model accounting also for functional limitations, those reporting joint pain or arthritis paid $1638 more than those without. Those with functional limitations regardless of comorbidity paid $3308 more than those without functional limitations, which was a higher marginal impact than any other comorbidity included in the model.

**Table 2:** Sequential explanatory model for marginal total healthcare expenditures in those with self-reported joint pain and/or arthritis

Variable	Model 1	Model 2	Model 3	Model 4	Model 5
No reported joint pain and/or arthritis (reference)	–	–	–	–	–
Self-reported joint pain and/or arthritis	$6773^*^	$4427^*^	$3980^*^	$2764^*^	$1638^*^
Predisposing factors					
Age, years					
18–24 (reference)		–	–	–	–
25–44		$1571^*^	$1873^*^	$2381^*^	$2372^*^
45–64		$3633^*^	$3717^*^	$3224^*^	$3038^*^
65+		$6150^*^	$3510^*^	$2087^*^	$1607^*^
Race					
Non-Hispanic white (reference)		–	–	–	–
Non-Hispanic black		−$883^**^	−$1053^**^	−$1325^**^	−$1287^*^
Non-Hispanic other		−$543	−$448	−$741	−$136
Hispanic		−$2196^*^	−$1960^*^	−$1845^*^	−$1683^*^
Sex					
Male (reference)		–	–	–	–
Female		$1053^*^	$929^*^	$1272^*^	$1383^*^
Region					
Northeast (reference)		–	–	–	–
Midwest		−$149	$78	−$101	−$168.78
South		−$514	−$69	−$458	−$325.85
West		$272	$489	$610	$746.14
MSA					
Rural (reference)		–	–	–	–
Urban		$797^**^	$1124^*^	$1216^*^	$1241^*^
Enabling factors					
Income					
< $25,000 (reference)			–	–	–
$25,000–$49,999			−$414	−$293	−$44
$50,000–$74,999			$3	$444	$817
> $75,000			$364	$1487^***^	$1761^**^
Employment					
Not employed (reference)			–	–	–
Employed			−$3239	−$2654^*^	−$2018^*^
Education					
Less than high school (reference)			–	–	–
High school			$849^***^	$1566^**^	$1634^*^
College			$709	$1242^*^	$1305^*^
Graduate school			$1515^**^	$2493^*^	$2457^*^
Insurance					
Any private (reference)			–	–	–
Public only			$1526^***^	$1639 ^***^	$947
No insurance			−$3615^*^	−$3875^*^	−$3943^*^
Marital status					
Married (reference)			–	–	–
Separated/divorced/widowed			$63	$34	−$131
Never married			−$55	$79	−$172
Need factors^****^					
Asthma				$2428^*^	$2001^*^
Diabetes				$3001^*^	$2669^*^
Emphysema				$2476^***^	$1559^***^
High blood pressure				$938^*^	$876^*^
High cholesterol				$1149^*^	$1194^*^
Stroke				$2683^*^	$1727^***^
Depression				$3182^*^	$1939^*^
CVD				$2814^*^	$2375^*^
BMI					
Underweight (reference)				–	–
Normal				$726	$1200
Overweight				$388	$732
Obese				$534	$824
Risk factor of interest^****^					
Any limitations					$3308^*^
IADL					$2488^***^
ADL					$2284^***^
Walk limitations					$253

*Abbreviations: ADL* Activities of daily living, *BMI* Body mass index, *CVD* Cardiovascular disease, *IADL* Instrumental activities of daily living, *MSA* Metropolitan statistical area

Sequential linear models were used, with each column representing a separate generalized linear model using gamma distribution

^*^ *p* < 0.001

^**^ *p* < 0.01

^***^ *p* < 0.05

^****^Reference groups compared with those without need factor or risk factor

## Discussion

Our analyses showed that individuals self-reporting any diagnosis of arthritis and/or joint pain had substantially higher mean total healthcare expenditures ($6773) than individuals without either. In addition, whereas accounting for predisposing, enabling, and need variables lowered this marginal difference in expenditures, it did not remove the significance. Adjusting for functional limitations further decreased the difference in healthcare expenditures to $1638, but it did not remove significance. The marginal decrease resulting from adding functional limitations into the model was similar in size to the marginal decrease resulting from adding all other comorbidities.

This study emphasizes the need for aggressive measures and strategies for the prevention, early recognition, and treatment of functional limitations in populations with arthritis and joint pain because they have significant impact on differences in medical expenditures. In an investigation of risk factors for work disability, Allaire et al. [17] found that two of the most common indicators of work cessation were severity of disease condition and physical limitations in arthritis and other rheumatic conditions. Work cessation is especially important to populations with chronic conditions, often manifested in two primary concerns. First, loss of employment means loss of income, and for a population that experiences disproportionately high healthcare costs, losing the primary source of income could lead many individuals to financial ruin. Second, according to the Henry J. Kaiser Family Foundation, 49% of the U.S. adult population obtain health insurance through their employers [18]. Thus, many individuals with arthritis or joint pain who cease working because of functional limitations will lose their health insurance and have to pay significant out-of-pocket expenses or be forced to purchase private insurance, which may be more expensive and/or provide less coverage.

Our findings are consistent with existing literature that estimates annual costs associated with some rheumatic conditions to be $10,000–$50,000 more per patient than those for the general population [19–22]. Major cost drivers include inpatient hospitalizations [23], poor physical and mental health, and low education and employment levels. Significant functional and emotional challenges resulting from symptoms, side effects, and complications may also include anxiety, depression, mood disorders, and decreased health-related quality of life [24–27], which further increase service use costs. In another study, Ozaras et al. [28] observed that in patients with ankylosing spondylitis and psoriatic arthritis, disease activity (self-reported pain and symptoms) was associated with greater physical limitation. Given the direct relationships observed between disease activity, comorbidities, and limitations [17, 28–31], the provision of optimal care to individuals with arthritis and joint pain is critical. This ultimately results in control of disease and slows the progression of related complications such as functional, activity, and sensory limitations.

Though the present study had a number of strengths, including the generalizability of results to the U.S. population, it has some limitations. First, diagnoses were based on self-report, which may differ from physician diagnosis. Second, though we attempted to include factors that could affect the relationship based on a theoretical framework, there are additional factors not in the dataset, including social determinants such as social support and emotional distress. Additionally, self-reports are subject to recall bias, which can introduce random error into the data. Finally, the data are cross-sectional in nature, which precludes commentary on causation or direction of the association.

## Conclusions

Our study shows that self-reported arthritis and/or joint pain of any type is associated with higher total healthcare expenditures, and though accounting for functional limitations decreases this difference, the difference remains significant. The growing economic and public health burden of arthritis and joint pain [17, 32, 33], as well as the corresponding complications of functional, activity, and sensory limitations, calls for an interdisciplinary approach and heightened awareness among providers to identify strategies that meet the needs of high-risk patients in order to prevent and delay disease progression. This study provides an estimate for potential savings from future interventions geared toward reduction of any limitations in patients with arthritis and joint pain. Future researchers may benefit from this analysis and expand upon it by examining specifically where these increased costs are coming from, such as whether the increased cost is due to a greater number of visits to health professionals, in-hospital treatments, medications, or other causes. Enumerating the breakdown of these expenditures would allow future development of solutions to target specific areas with disproportionately high costs.

## Abbreviations

ADL: Activities of daily living
BMI: Body mass index
CVD: Cardiovascular disease
GLM: Generalized linear model
IADL: Instrumental activities of daily living
MEPS: Medical Expenditures Panel Survey
MSA: Metropolitan statistical area
NHW: Non-Hispanic white

## References

[1] Ma VY, Chan L, Carruthers KJ (2014). Incidence, prevalence, costs, and impact on disability of common conditions requiring rehabilitation in the United States: stroke, spinal cord injury, traumatic brain injury, multiple sclerosis, osteoarthritis, rheumatoid arthritis, limb loss, and back pain. Arch Phys Med Rehabil.

[2] Spaetgens B, Tran-Duy A, Wijnands JM, van der Linden S, Boonen A (2015). Health and utilities in patients with gout under the care of a rheumatologist. Arthritis Care Res (Hoboken).

[3] Vuong TD, Wei F, Beverly CJ (2015). Absenteeism due to functional limitations caused by seven common chronic diseases in US workers. J Occup Environ Med.

[4] Theis KA, Murphy L, Hootman JM, Wilkie R (2013). Social participation restriction among US adults with arthritis: a population-based study using the International Classification of Functioning, Disability and Health. Arthritis Care Res (Hoboken).

[5] Durcan L, Wilson F, Conway R, Cunnane G, O’Shea FD (2012). Increased body mass index in ankylosing spondylitis is associated with greater burden of symptoms and poor perceptions of the benefits of exercise. J Rheumatol.

[6] Iqbal I, Dasgupta B, Taylor P, Heron L, Pilling C (2012). Elicitation of health state utilities associated with differing durations of morning stiffness in rheumatoid arthritis. J Med Econ.

[7] Nuñez DE, Keller C, Ananian CD (2009). A review of the efficacy of the self-management model on health outcomes in community-residing older adults with arthritis. Worldviews Evid Based Nurs.

[8] Turner J, Turner R (2004). Physical disability, unemployment, and mental health. Rehabil Psych.

[9] Ward MM (2002). Functional disability predicts total costs in patients with ankylosing spondylitis. Arthritis Rheum.

[10] Dall TM, Gallo P, Koenig L, Gu Q, Ruiz D (2013). Modeling the indirect economic implications of musculoskeletal disorders and treatment. Cost Eff Resour Alloc.

[11] Spaetgens B, Wijnands JM, van Durme C, van der Linden S, Boonen A (2015). Cost of illness and determinants of costs among patients with gout. J Rheumatol.

[12] Wolfe F, Michaud K, Choi HK, Williams R (2005). Household income and earnings losses among 6,396 persons with rheumatoid arthritis. J Rheumatol.

[13] Agency for Healthcare Research and Quality (AHRQ) (2013). MEPS HC-147: 2011 full year consolidated data file.

[14] Agency for Healthcare Research and Quality (AHRQ) (2013). MEPS HC-036: 1996–2011 pooled linkage variance estimation file.

[15] Agency for Healthcare Research and Quality (AHRQb). Medical Expenditure Panel Survey. 2011 Full year consolidated data file 2013c, Available from http://meps.ahrq.gov/mepsweb/data_stats/download_data_files.jsp.

[16] Aday LA, Andersen R (1974). A framework for the study of access to medical care. Health Serv Res.

[17] Allaire SJ, AlHeresh R, Keysor JJ (2013). Risk factors for work disability associated with arthritis and other rheumatic conditions. Work.

[18] Henry J, Kaiser Family Foundation (KFF) (2015). Health insurance coverage of the total population.

[19] Kan HJ, Song X, Johnson BH, Bechtel B, O’Sullivan D, Molta CT (2013). Healthcare utilization and costs of systemic lupus erythematosus in Medicaid. Biomed Res Int.

[20] Panopalis P, Yazdany J, Gillis JZ, Julian L, Trupin L, Hersh AO (2008). Health care costs and costs associated with changes in work productivity among persons with systemic lupus erythematosus. Arthritis Rheum.

[21] Panopalis P, Clarke AE, Yelin E (2012). The economic burden of systemic lupus erythematosus. Best Pract Res Clin Rheumatol.

[22] Birnbaum H, Pike C, Kaufman R, Marynchenko M, Kidolezi Y, Cifaldi M (2010). Societal cost of rheumatoid arthritis patients in the US. Curr Med Res Opin.

[23] Garris C, Jhingran P, Bass D, Engel-Nitz N, Riedel A, Dennis G (2013). Healthcare utilization and cost of systemic lupus erythematosus in a US managed care health plan. J Med Econ.

[24] Jolly M (2005). How does quality of life of patients with systemic lupus erythematosus compare with that of other common chronic illnesses?. J Rheumatol.

[25] Da Costa D, Dobkin PL, Fitzcharles MA, Fortin PR, Beaulieu A, Zummer M (2000). Determinants of health status in fibromyalgia: a comparative study with systemic lupus erythematosus. J Rheumatol.

[26] Greco C, Rudy T, Manzi S (2003). Adaptation to chronic pain in systemic lupus erythematosus: applicability of the multidimensional pain inventory. Pain Med.

[27] Cornwell C, Schmitt M (1990). Perceived health status, self-esteem and body image in women with rheumatoid arthritis or systemic lupus erythematosus. Res Nurs Health.

[28] Ozaras N, Havan N, Poyraz E, Rezvani A, Aydin T (2016). Functional limitations due to foot involvement in spondyloarthritis. J Phys Ther Sci.

[29] Omariba DWR (2011). Gender differences in functional limitations among Canadians with arthritis: the role of disease duration and comorbidity. Health Rep.

[30] Marques WV, Cruz VA, Rego J, Silva NA (2016). The impact of comorbidities on the physical function in patients with rheumatoid arthritis. Rev Bras Reumatol Engl Ed.

[31] Shih VC, Song J, Chang RW, Dunlop DD (2005). Racial differences in activities of daily living limitation onset in older adults with arthritis: a national cohort study. Arch Phys Med Rehab.

[32] Drenkard C, Bao G, Dennis G, Kan HJ, Jhingran PM, Molta CT (2014). Burden of systemic lupus erythematosus on employment and work productivity: data from a large cohort in the southeastern United States. Arthritis Care Res (Hoboken).

[33] Looper KJ, Mustafa SS, Zelkowitz P, Purden M, Baron M, McGill Early Arthritis Research Group (2012). Work instability and financial loss in early inflammatory arthritis. Int J Rheum Dis.

